# Sex-specific and metabolic subgroup heterogeneity in high-density lipoprotein cholesterol associations with diabetic kidney disease risk: a retrospective cohort study

**DOI:** 10.1186/s12944-025-02632-4

**Published:** 2025-06-07

**Authors:** Huabin Wang, Xuanlin Jin, Fenfang Lin, Guangming Chen, Meili Lin, Yongjun Ma

**Affiliations:** 1 Department of Clinical Laboratory, Affiliated Jinhua Hospital, Zhejiang University School of Medicine, Renming Road, Jinhua city, 321000 Zhejiang Province China; 2 Wenzhou Medical University, Wenzhou, 325035 Zhejiang China; 3 Department of General Practice, Qiubin Community Health Service Center, Jinhua, 321016 Zhejiang China; 4 Department of General Practice, Affiliated Jinhua Hospital, Zhejiang University School of Medicine, Jinhua, 321000 Zhejiang China

**Keywords:** Diabetic kidney disease, Dual effects, High-density lipoprotein cholesterol, Metabolic context-dependent, Subgroup heterogeneity

## Abstract

**Background:**

The role of high-density lipoprotein cholesterol (HDL-C) in diabetic kidney disease (DKD) remains controversial. This study aimed to delineate the subgroup-specific relationships between the two by exploring cumulative and threshold effects.

**Methods:**

3,040 patients with type 2 diabetes and no baseline evidence of DKD were included. Cox proportional hazards regression models were performed to investigate the potential relationship between HDL-C level and DKD risk. To address subgroup heterogeneity, sex-stratified restricted cubic splines (RCS) were employed to model nonlinear relationships. The optimal threshold was identified through the maximum selected statistics and validated via 1,000 bootstrap iterations. Subgroup analyses stratified by sex, diabetes duration, and metabolic status were performed to evaluate heterogeneity. Survival analysis using Kaplan-Meier curves further validated these threshold effects.

**Results:**

During a median follow-up of 3.13 years, 665 subjects (21.9%) progressed to DKD. Overall, each 1 mmol/L increase in HDL-C level independently reduced DKD risk by 43%. RCS analysis demonstrated an inverse correlation between HDL-C and DKD risk (*P* for overall = 0.025, *P* for nonlinear = 0.317), with increased risk reduction at lower concentrations, plateauing at higher levels. A robust threshold of 0.93 mmol/L was identified, showing significantly stronger protection against DKD progression (hazard ratio (HR) = 0.69, *P* < 0.001) compared to the traditional cutoff (HR = 0.86, *P* = 0.109). Females showed continuous protection (HR = 0.41, *P* = 0.009) without threshold dependency. The male and diabetes duration < 10 years subgroups exhibited threshold effects at > 0.93 mmol/L without continuous protection. The metabolically unstable (hypertension, poorly controlled glycemia, body mass index (BMI) > 28 kg/m^2^) and BMI < 24 kg/m² subgroups displayed dual effects (*P <* 0.05). Survival analysis confirmed lower cumulative DKD incidence with HDL-C > 0.93 mmol/L (*P* = 0.007).

**Conclusions:**

This study reveals sex- and metabolic context-dependent heterogeneity in HDL-C-DKD associations: males and short-duration diabetes exhibited threshold effects (0.93 mmol/L), females showed continuous protection, and subgroups with hypertension, poorly controlled glycemia, or obesity (BMI > 28 kg/m²) exhibited both continuous protection and threshold effects. These findings may inform individualized risk stratification in specific populations.

**Supplementary Information:**

The online version contains supplementary material available at 10.1186/s12944-025-02632-4.

## Background

Diabetic kidney disease (DKD) is a common complication of diabetes [1, 2]. The global number of individuals with diabetes continues to increase, with projections exceeding 783 million by 2045 [3]. Approximately 20–40% of individuals with type 2 diabetes (T2D) develop DKD [4], posing a major global health burden. DKD not only significantly increases the risk of renal failure but is also closely linked to cardiovascular diseases [5], contributing to higher mortality rates and diminished quality of life in individuals with diabetes [6]. Early assessment of DKD risk and effective management of risk factors are critical for delaying kidney function decline and reducing related complications.

High-density lipoprotein cholesterol (HDL-C) is widely recognized as a protective component of lipid metabolism that plays essential roles in removing excess cholesterol, maintaining vascular endothelial function, and regulating inflammation and oxidative stress [7]. Recent studies have suggested that it may slow DKD progression by modulating the renal microenvironment [8]; however, its protective role in DKD remains unclear. Some studies indicate that higher HDL-C levels reduce DKD risk [9–11], with a dose-response relationship (6% risk reduction per 10 mg/dL increase in HDL-C; hazard ratio (HR) = 0.94, 95% confidence interval (CI): 0.92–0.96) [11], while others found no significant association [12] or a U-shaped relationship in which risk progression increased in the lowest and highest deciles of HDL-C [13, 14]. The HDL-C–DKD relationship is hypothesized to be not solely a linear or threshold effect but rather a dynamic process influenced by multiple factors, with its protective effects potentially varying across clinical subgroups (including sex, diabetes duration, and metabolic status).

This longitudinal observational study systematically investigated whether threshold effects or nonlinear relationships existed between HDL-C concentrations and the DKD risk, and evaluated whether these associations differed according to sex, diabetes duration, or metabolic status. These findings are expected to elucidate the relationship between HDL-C and DKD risk and provide a theoretical basis for individualized DKD prevention and management strategies.

## Methods

### Study population

**Fig. 1:**
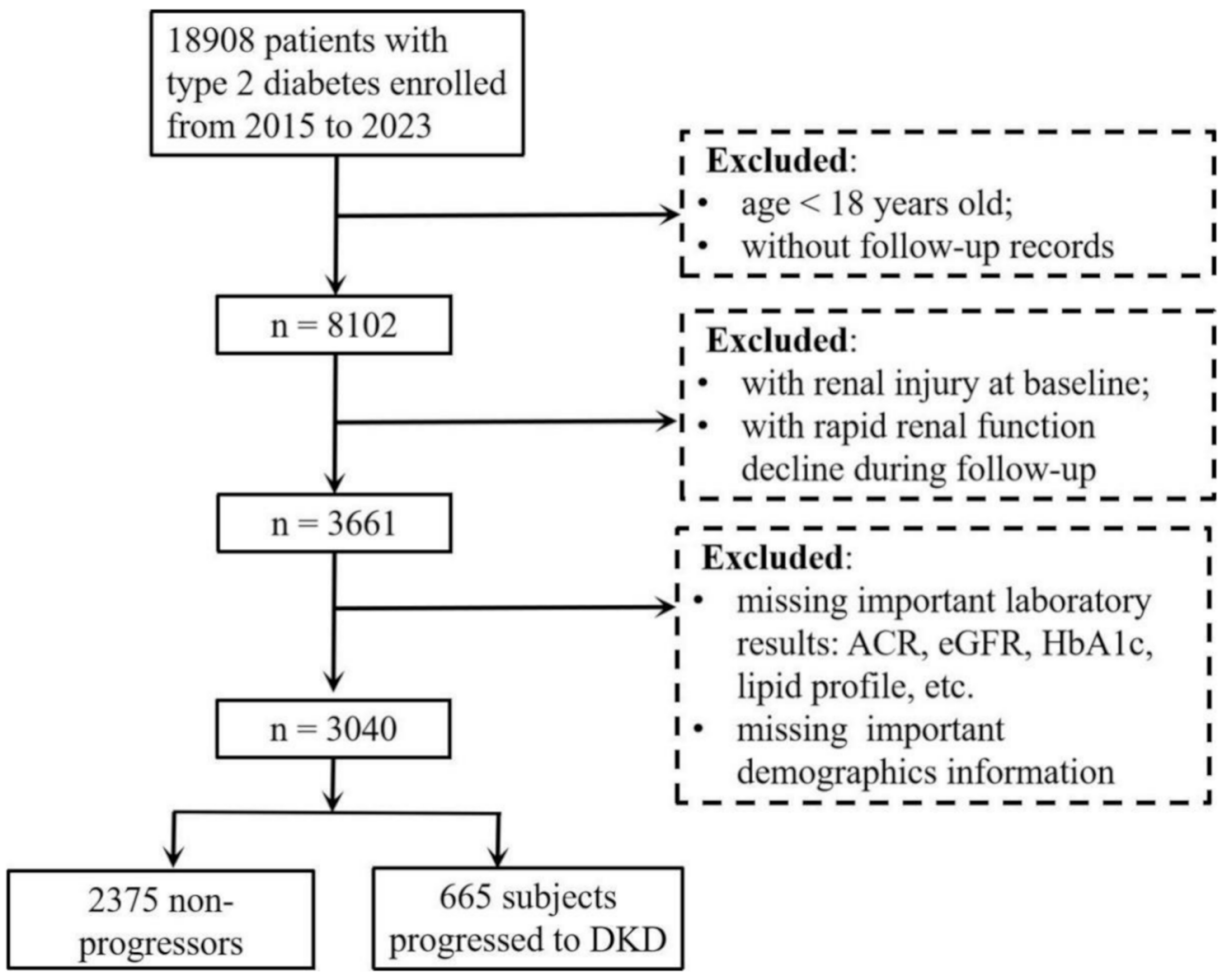
The participant flow chart. eGFR: estimated glomerular filtration rate; ACR: albumin-to-creatinine ratio; DKD: diabetic kidney disease; HbA1c: glycated hemoglobin

This retrospective cohort study included 3,040 patients with type 2 diabetes who did not have DKD at baseline. Participants were selected from the electronic medical records of the Affiliated Jinhua Hospital, Zhejiang University School of Medicine, between 2015 and 2023. The inclusion criteria were a diagnosis of type 2 diabetes [15]; age > 18 years; absence of renal dysfunction at baseline, defined by an albumin-to-creatinine ratio (ACR) < 30 mg/g and/or estimated glomerular filtration rate (eGFR) > 60 mL/min/1.73 m²; and at least one follow-up assessment of kidney function per year during the study period. The exclusion criteria were subjects missing critical clinical data; with rapid renal function decline during follow-up, defined as an acute kidney injury event according to KDIGO criteria (≥ 50% increase in serum creatinine within 7 days or ≥ 0.3 mg/dL absolute increase); or diagnosed with other types of kidney disease (glomerulonephritis, polycystic kidney disease). The details of participant enrollment are shown in Fig. 1. This study was approved by the Ethics Committee of the Affiliated Jinhua Hospital, Zhejiang University School of Medicine (ethical approval number: (Res) 2024-Ethical Review-sb58).

### Data collection

The clinical data were retrieved from the institution’s electronic health records database. Baseline clinical characteristics, including demographics, comorbidities, laboratory results, and medication use, were recorded. Key variables included age, sex, duration of diabetes, hypertension status, body mass index (BMI), glycated hemoglobin (HbA1c) levels, uric acid, renal function parameters (ACR, eGFR, serum creatinine), lipid profile (HDL-C, low-density lipoprotein cholesterol (LDL-C), triglycerides (TG), and total cholesterol (TC)), and the use of medications (angiotensin-converting enzyme inhibitor (ACEI), angiotensin receptor blocker (ARB), insulin, lipid-lowering drugs such as fibrate or statin, SGLT2 inhibitors (SGLT2is) and GLP-1 receptor agonists (GLP-1RAs). Follow-up data on kidney function, specifically ACR and eGFR, were collected annually for up to 8 years.

### Definitions

DKD was defined as ACR ≥ 30 mg/g or a decrease in eGFR < 60 mL/min/1.73 m² during follow-up. eGFR was calculated using the Xiangya equation [16], a validated formula for Chinese populations. Glycemic control status was dichotomized based on the American Diabetes Association target [17], with HbA1c > 7.0% indicating poor control. Individuals were grouped into normal weight (BMI < 24 kg/m^2^), overweight (24–28 kg/m^2^), and obese (> 28 kg/m^2^) based on standardized BMI classifications [18]. Age was stratified at 60 years while diabetes duration was dichotomized at 10 years, a recognized inflection point for accelerated microvascular complications [19]. In Cox regression analyses, covariates were selected through a hypothesis-driven approach: Model 1 incorporated adjustments for age and sex; Model 2 extended these adjustments to include systolic blood pressure (SBP), BMI, TG, LDL-C, HbA1c, TC, baseline ACR, and serum creatinine; and Model 3 expanded upon Model 2 by adding hypertension status, diabetes duration, and SGLT2is/GLP-1RAs, ensuring adjustment for variables with significant univariate associations (*P <* 0.05) or established roles in DKD pathogenesis [20–22].

### Statistical analysis

Baseline characteristics of participants were analyzed using descriptive methods, with categorical data reported as counts (percentages) and continuous measures summarized as mean values ± standard deviations. Comparisons between patients progressing to DKD and non-progressors utilized the chi-square test for categorical variables, while continuous measures were analyzed with either the Student’s t-test or Mann-Whitney U test, depending on data distribution. Cox proportional hazard regression models were used to assess the relationship between HDL-C levels and DKD risk. HR with 95% CIs were calculated for HDL-C as a continuous and categorical variable. The potential nonlinear relationship was explored using Cox regression models with restricted cubic splines (RCS). Additionally, maximum selected statistics (MSS) and bootstrap resampling (1,000 iterations) were applied to determine and validate the optimal HDL-C threshold for predicting the risk of DKD. Sensitivity analyses compared the MSS-derived threshold with the guideline-recommended cutoffs (1.0 mmol/L). Subgroup analyses were performed based on sex, diabetes duration, and metabolic status (hypertension, glycemic control, and BMI) to evaluate potential heterogeneity. Kaplan-Meier methodology was employed to estimate survival probabilities, with between-group differences evaluated via log-rank testing. In this study, multiple imputations were not required because missing values were excluded. Statistical analyses were all conducted in R (v3.6.3) and SPSS (v26.0), applying a significance threshold of *P* < 0.05.

## Results

### Baseline clinical characteristics by DKD progression status

Table 1 summarized the characteristics of the 3,040 subjects, stratified by their progression status for DKD. The mean age was 58.04 ± 11.55 years, with 1,163 females (38.2%). The median diabetes duration at baseline was 6.0 years, with 38.1% (*n* = 1,156) having a diabetes duration > 10 years and 61.9% (*n* = 1,884) having a duration of < 10 years. During a median follow-up of 3.13 years, 665 subjects (21.9%) progressed to DKD. Compared to non-progressors, those who progressed to DKD were older (60.7 vs. 57.3 years; *P <* 0.001), and had a longer diabetes duration (8 vs. 6 years, *P <* 0.001), higher HbA1c levels (8.76% vs. 8.24%, *P <* 0.001), and significantly higher baseline ACR (14.67 vs. 9.00 mg/g, *P <* 0.001). Additionally, DKD progressors had a higher proportion of hypertension (58.4% vs. 46.9%, *P <* 0.001), insulin use (43.1% vs. 31.0%, *P <* 0.001), and ACEI/ARB use (22.2% vs. 18.1%, *P =* 0.017) and a lower usage rate of SGLT2is/GLP-1RAs (8.7% vs. 11.7%, *P =* 0.026). HDL-C levels were marginally lower in progressors (1.11 vs. 1.13 mmol/L, *P =* 0.040).

**Table 1:** Baseline characteristics of the study population stratified by DKD progression

	Overall	Non-progressors	Progressed to DKD	*P* value
	*n* = 3040	*n* = 2375	*n* = 665	
Age, year	58.04 ± 11.55	57.29 ± 11.26	60.70 ± 12.18	< 0.001
Female	1163 (38.2)	878 (36.9)	285 (42.8)	0.006
Hypertension	1505 (49.5)	1116 (46.9)	389 (58.4)	< 0.001
History of drinking	1054 (34.6)	820 (34.5)	234 (35.1)	0.751
History of smoking	1111 (36.5)	867 (36.5)	244 (36.6)	0.930
Diabetic duration, year	6 (2, 10)	6 (2, 10)	8 (4, 13)	< 0.001
Follow up time, year	3.13 (1.72, 4.88)	3.22 (1.80, 4.97)	2.97 (1.55, 4.72)	0.008
SBP, mmHg	133.60 ± 18.23	132.84 ± 17.82	136.31 ± 19.39	< 0.001
DBP, mmHg	78.52 ± 11.28	78.74 ± 11.21	77.75 ± 11.51	0.050
BMI, kg/m^2^	24.60 ± 4.26	24.62 ± 4.17	24.53 ± 4.55	0.090
HbA1c, %	8.36 ± 2.25	8.24 ± 2.21	8.76 ± 2.35	< 0.001
TC, mmol/l	4.38 ± 1.16	4.40 ± 1.13	4.29 ± 1.27	0.034
LDL-C, mmol/l	2.84 ± 0.84	2.85 ± 0.82	2.80 ± 0.91	0.115
HDL-C, mmol/l	1.13 ± 0.30	1.13 ± 0.31	1.11 ± 0.29	0.040
TG, mmol/l	1.45 (1.02, 2.14)	1.46 (1.03, 2.14)	1.42 (1.01, 2.16)	0.879
FBG, mmol/l	7.68 ± 2.84	7.57 ± 2.76	8.07 ± 3.05	< 0.001
UA, µmol/L	313.51 ± 88.21	312.98 ± 85.73	315.41 ± 96.59	0.531
Serum creatinine, µmol/L	74.39 ± 14.09	74.11 ± 13.57	75.38 ± 15.76	0.040
eGFR, ml/min/1.73 m^2^	78.14 ± 9.05	78.62 ± 8.83	76.43 ± 9.61	< 0.001
ACR, mg/g	10.01 (4.98, 16.40)	9.00 (4.25, 14.48)	14.67 (8.8, 21.5)	< 0.001
Insulin therapy	1025 (33.7)	738 (31.0)	287 (43.1)	< 0.001
ACEI/ARB use	579 (19.0)	431 (18.1)	148 (22.2)	0.017
Fibrate/statin use	508 (16.7)	390 (16.4)	118 (17.7)	0.419
SGLT2i/GLP-1RA use	338 (11.1)	280 (11.7)	58 (8.7)	0.026

DKD: diabetic kidney disease; HDL-C: high-density lipoprotein cholesterol; SBP: systolic blood pressure; DBP: diastolic blood pressure; BMI: body mass index; HbA1c: glycated hemoglobin; TC: total cholesterol; LDL-C: low-density lipoprotein cholesterol; TG: triglycerides; FBG: fasting blood glucose; UA: uric acid; eGFR: estimated glomerular filtration rate; ACR: albumin-to-creatinine ratio; ACEI: angiotensin-converting enzyme inhibitors; ARB: angiotensin receptor blockers; SGLT2i: sodium-glucose cotransporter-2 inhibitors; GLP-1RA: glucagon-like peptide-1 receptor agonists

### Association between HDL-C levels and DKD risk in the overall cohort

In the adjusted models (Table 2), each 1 mmol/L increase in HDL-C significantly reduced DKD risk in Models 1 (HR = 0.67, 95% CI: 0.51–0.89, *P =* 0.006) and 2 (HR = 0.61, 95% CI: 0.42–0.94, *P =* 0.024). After full adjustment for confounders (Model 3), the risk reduction increased to 43% (HR = 0.57 [0.37–0.87], *P =* 0.010), indicating that higher HDL-C levels were an independent protective factor for DKD risk. When HDL-C was grouped by quartiles, Q2, Q3, and Q4 significantly reduced DKD risk compared to Q1 (lowest levels) (all *P <* 0.05), with Q4 showing a 37% reduction (HR = 0.63 [0.47–0.85], *P =* 0.002). However, the risk reduction in Q3 was weaker than in Q2 (0.67 vs. 0.78) in the three models, suggesting that the protective effect might not strictly follow a linear gradient, indicating a potential non-linear relationship or threshold effect between HDL-C and DKD risk.

**Table 2:** Cox regression models for the association of HDL-C levels with DKD onset risk

	Model 1			Model 2			Model 3		
HDL-C level	HR	95%CI	*P* value	HR	95%CI	*P* value	HR	95%CI	*P* value
per unit increase	0.67	0.51–0.89	0.006	0.61	0.42–0.94	0.024	0.57	0.37–0.87	0.010
Quartile 1	Ref			Ref			Ref		
Quartile 2	0.68	0.55–0.84	< 0.001	0.68	0.55–0.84	< 0.001	0.67	0.54–0.84	< 0.001
Quartile 3	0.77	0.62–0.95	0.016	0.78	0.62–1.00	0.048	0.78	0.61–0.98	0.035
Quartile 4	0.67	0.54–0.84	< 0.001	0.66	0.49–0.89	0.006	0.63	0.47–0.85	0.002
*P* for trend	< 0.001			0.003			0.002		

DKD: diabetic kidney disease; HDL-C: high-density lipoprotein cholesterol. HR: hazard ratios; Quartile 1 of HDL-C: ≤ 0.92 mmol/L; Quartile 2 of HDL-C: 0.93–1.09 mmol/L; Quartile 3 of HDL-C: 1.10–1.28 mmol/L; Quartile 4 of HDL-C: > 1.28 mmol/L

### RCS analysis of HDL-C and DKD risks and optimal threshold determination

**Fig. 2:**
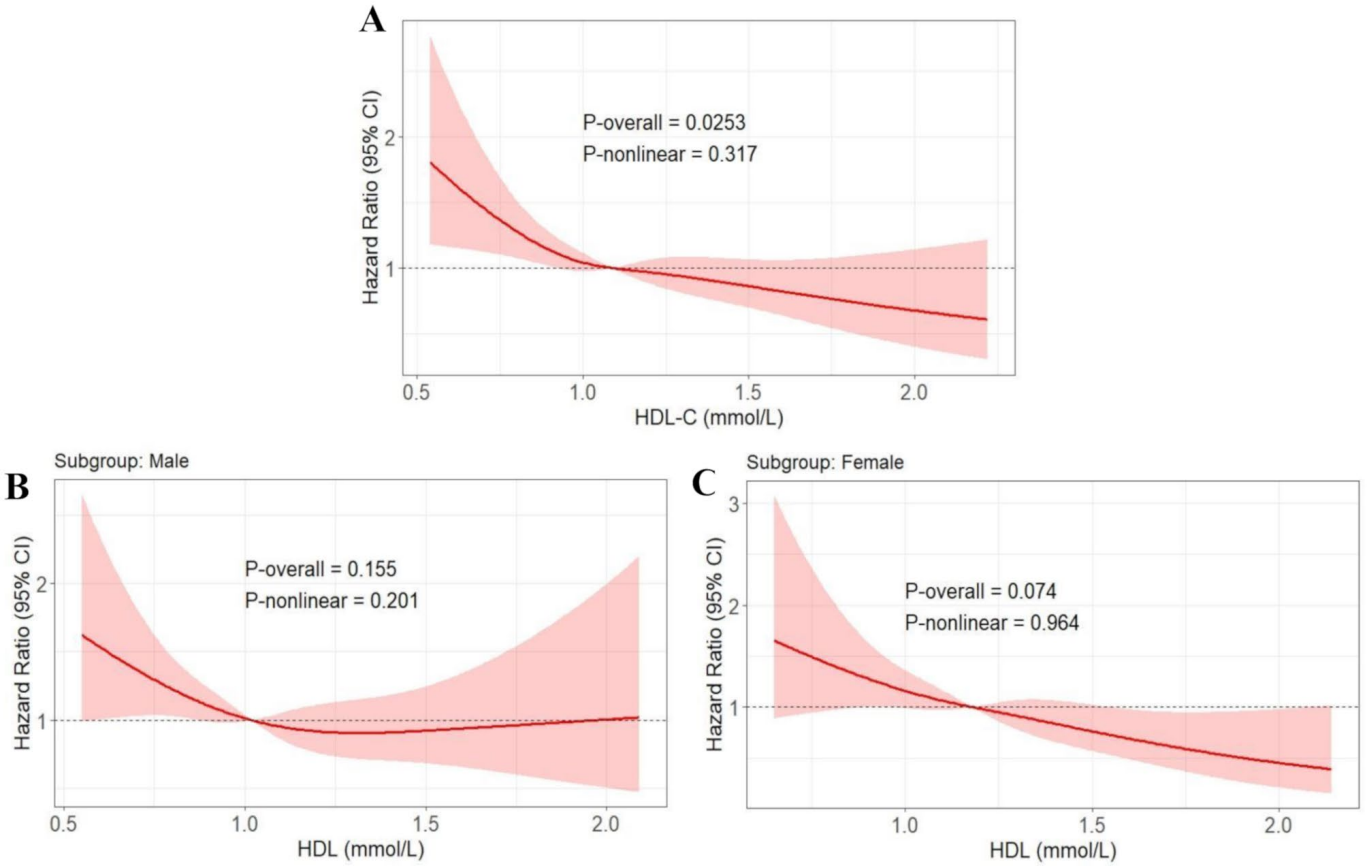
Association between HDL-C levels and DKD risk analyzed via RCS with Cox proportional hazards regression adjusted for age, sex, SBP, BMI, TG, LDL-C, HbA1c, TC, baseline ACR, and serum creatinine, hypertension status, diabetes duration, and SGLT2i/GLP-1RA use in the overall cohort (**A**), male subgroup (**B**) and female subgroup (**C**). DKD: diabetic kidney disease; RCS: restricted cubic spline; HDL-C: high-density lipoprotein cholesterol; SBP: systolic blood pressure; BMI: body mass index; HbA1c: glycated hemoglobin; TC: total cholesterol; LDL-C: low-density lipoprotein cholesterol; TG: triglycerides; ACR: albumin-to-creatinine ratio; SGLT2i: sodium-glucose cotransporter-2 inhibitors; GLP-1RA: glucagon-like peptide-1 receptor agonists

**Fig. 3:**
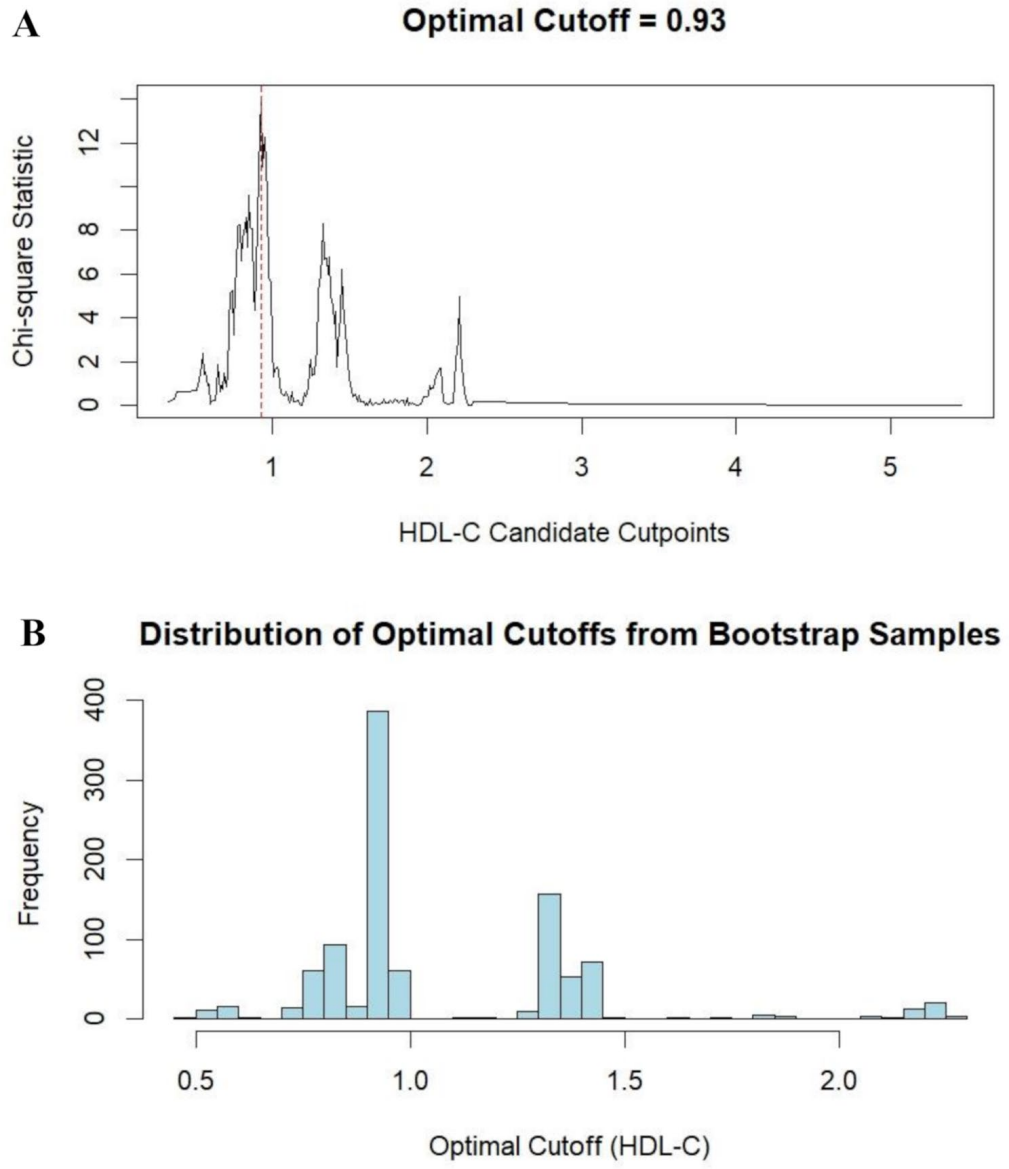
Determination of the optimal HDL-C threshold using the MSS method (**A**) and bootstrap validation (**B**) in the overall cohort. MSS: maximum selected statistics; HDL-C: high-density lipoprotein cholesterol

After adjusting for Model 3 covariates, RCS analysis showed a significant negative correlation between HDL-C levels and DKD risk (*P =* 0.025). Although the nonlinearity test was not significant (*P* for nonlinear = 0.317), the slope of risk reduction was steeper at lower HDL-C concentrations and plateaued at higher levels, suggesting a potential threshold effect (Fig. 2A). MSS determined the optimal threshold for HDL-C to be 0.93 mmol/L (Fig. 3A). Bootstrap internal validation further validated the robustness of this threshold (Fig. 3B), with the cutoff predominantly clustered within 0.90–0.95 mmol/L (frequency ≈ 400) despite a wide 95% CI (0.60–2.20 mmol/L).

Given the sex-specific heterogeneity of the biological effects of HDL-C, RCS, and MSS analyses stratified by sex were performed. The RCS analysis revealed a linear inverse correlation between HDL-C levels and DKD risk in the female subgroup, whereas an L-shaped curve relationship was observed in the male subgroup (Fig. 2B and C), although both *P* for overall and *P* for nonlinear showed no statistical significance for either. In the male subgroup, the optimal threshold for HDL-C was also identified as 0.93 mmol/L. As shown in Supplementary Figure S1, bootstrap clustering analysis confirmed the consistent stability of the HDL-C threshold (0.93 mmol/L) in males, with the cutoff predominantly clustered within 0.90–0.95 mmol/L (frequency > 700). However, in the female subgroup, although the MSS method identified an HDL-C threshold of 1.32 mmol/L (Supplementary Figure S2A), bootstrap validation revealed substantial instability in this cutoff (Supplementary Figure S2B). The distribution of bootstrap-derived thresholds spanned a wide range (95% CI: 0.62–1.96 mmol/L), with only approximately 15% of iterations clustering near 1.30–1.35 mmol/L (frequency ≈ 150), indicating that no robust HDL-C cutoff existed for DKD risk prediction in females and suggesting a likely absence of threshold-dependent effects in this subgroup.

### Subgroup heterogeneity in HDL-C and DKD risk associations

**Fig. 4:**
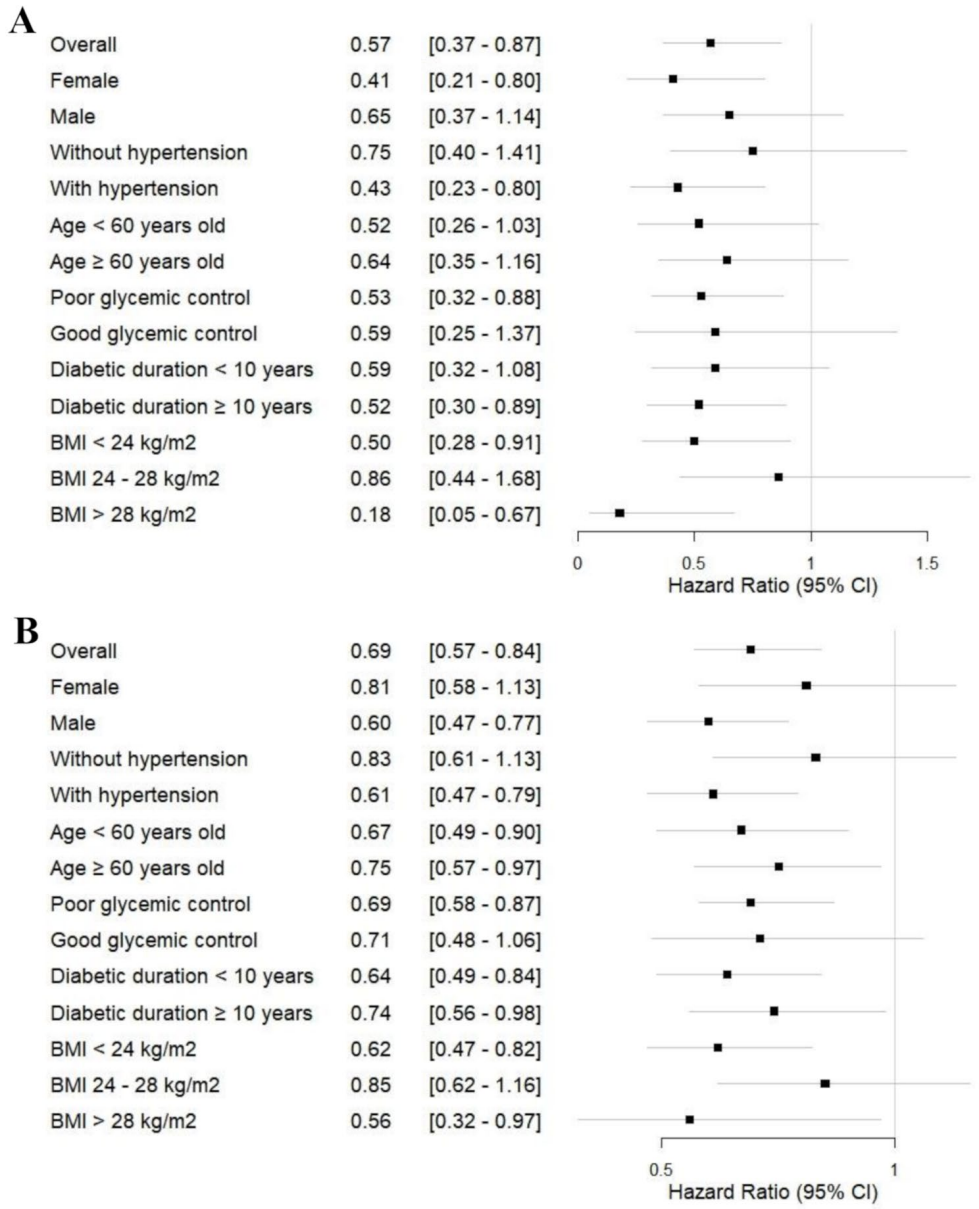
Subgroup analyses of HDL-C as a continuous variable (**A**) and as a threshold-defined (0.93 mmol/L) categorical variable (**B**) in relation to DKD risk across clinical subgroups showed significant heterogeneity. DKD: diabetic kidney disease; HDL-C: high-density lipoprotein cholesterol; BMI: body mass index

Subgroup analyses revealed substantial heterogeneity in HDL-C-DKD risk associations after adjusting for Model 3 covariates (Figs. 4 A-B). Sex-specific patterns emerged, with male participants exhibiting a robust threshold effect at HDL-C ≥ 0.93 mmol/L (HR = 0.60 [0.47–0.77], *P <* 0.001), aligning with the MSS-derived cutoff validated by tightly clustered bootstrap thresholds. In contrast, female participants demonstrated a continuous protective gradient (HR = 0.41 per 1 mmol/L, *P* = 0.009) rather than threshold dependency, consistent with the absence of a statistically significant MSS threshold and linear RCS trend. In the group with diabetes duration < 10 years, HDL-C as a categorical variable (with 0.93 mmol/L as the cutoff) showed a significant threshold effect, with HDL-C ≥ 0.93 mmol/L significantly lowering DKD risk (HR = 0.64 [0.39–0.84], *P =* 0.001); no significant relationship was found when HDL-C was considered as a continuous variable.

In subgroups with hypertension, poor glycemic control, diabetes duration > 10 years, BMI > 28 kg/m², and BMI < 24 kg/m², HDL-C exhibited a continuous protective gradient (HR = 0.18–0.59, *P <* 0.05) and a significant threshold effect at 0.93 mmol/L (HR = 0.56–0.69, *P <* 0.001). Table 3 summarizes the heterogeneous HDL-C-DKD risk associations across key clinical subgroups. In the groups without hypertension, with good glycemic control, and with a BMI of 24–28 kg/m², neither HDL-C as a continuous variable nor as a categorical variable was significantly associated with DKD risk.

**Table 3:** Heterogeneous associations between HDL-C and DKD risk in key clinical subgroups

Subgroup	Effect Pattern	Association Results
Female	Continuous	Each 1 mmol/L increase in HDL-C reduced DKD risk by 59% (HR = 0.41, *P* = 0.009)
Male	Threshold	HDL-C ≥ 0.93 mmol/L reduced DKD risk by 40% (HR = 0.60, *P* < 0.001).
With hypertension	Dual	Continuous: 57% risk reduction per 1 mmol/L (HR = 0.43, *P* = 0.007); Threshold: 39% reduction at ≥ 0.93 mmol/L (HR = 0.61, *P* < 0.001)
Poor glycemic control	Dual	Continuous: 47% risk reduction per 1 mmol/L (HR = 0.53, *P* = 0.014); Threshold: 31% reduction at ≥ 0.93 mmol/L (HR = 0.69, *P* = 0.001)
Diabetes duration < 10 years	Threshold	HDL-C ≥ 0.93 mmol/L reduced DKD risk by 36% (HR = 0.64, *P* = 0.001)
Diabetes duration ≥ 10 years	Dual	Continuous: 48% risk reduction per 1 mmol/L (HR = 0.52, *P* = 0.016); Threshold: 26% reduction at ≥ 0.93 mmol/L (HR = 0.74, *P* = 0.041)
BMI < 24 kg/m²	Dual	Continuous: 50% risk reduction per 1 mmol/L (HR = 0.50, *P* = 0.023); Threshold: 38% reduction at ≥ 0.93 mmol/L (HR = 0.62, *P* < 0.001)
BMI > 28 kg/m²	Dual	Continuous: 82% risk reduction per 1 mmol/L (HR = 0.18, *P* = 0.011); Threshold: 44% reduction at ≥ 0.93 mmol/L (HR = 0.56, *P* = 0.040)

DKD: diabetic kidney disease; HDL-C: high-density lipoprotein cholesterol; HR: hazard ratios; BMI: body mass index

### Sensitivity analysis of HDL-C threshold

To validate the existence of a threshold effect of HDL-C on DKD risk in specific subgroups, a sensitivity analysis using the traditional 1.0 mmol/L cutoff (Supplementary Figure S3) was performed. Compared to the 0.93 mmol/L cutoff (HR = 0.69, *P <* 0.001), the 1.0 mmol/L cutoff did not show a significant threshold effect in the overall cohort (HR = 0.86, *P =* 0.109) or the subgroup with poor glycemic control (HR = 0.86, *P =* 0.172). However, in males (HR = 0.51, *P <* 0.001), hypertensive individuals (HR = 0.61, *P <* 0.001), those with BMI < 24 kg/m² (HR = 0.62, *P =* 0.001), and those with BMI > 28 kg/m² (HR = 0.57, *P =* 0.046), HDL-C showed a threshold effect similar to that of the 0.93 mmol/L cutoff. Regardless of the cutoff value, HDL-C consistently demonstrated no significant threshold effect in females, suggesting that women might benefit from the cumulative effect of HDL-C rather than relying on a single threshold value.

### Survival analysis for validating the threshold effect

**Fig. 5:**
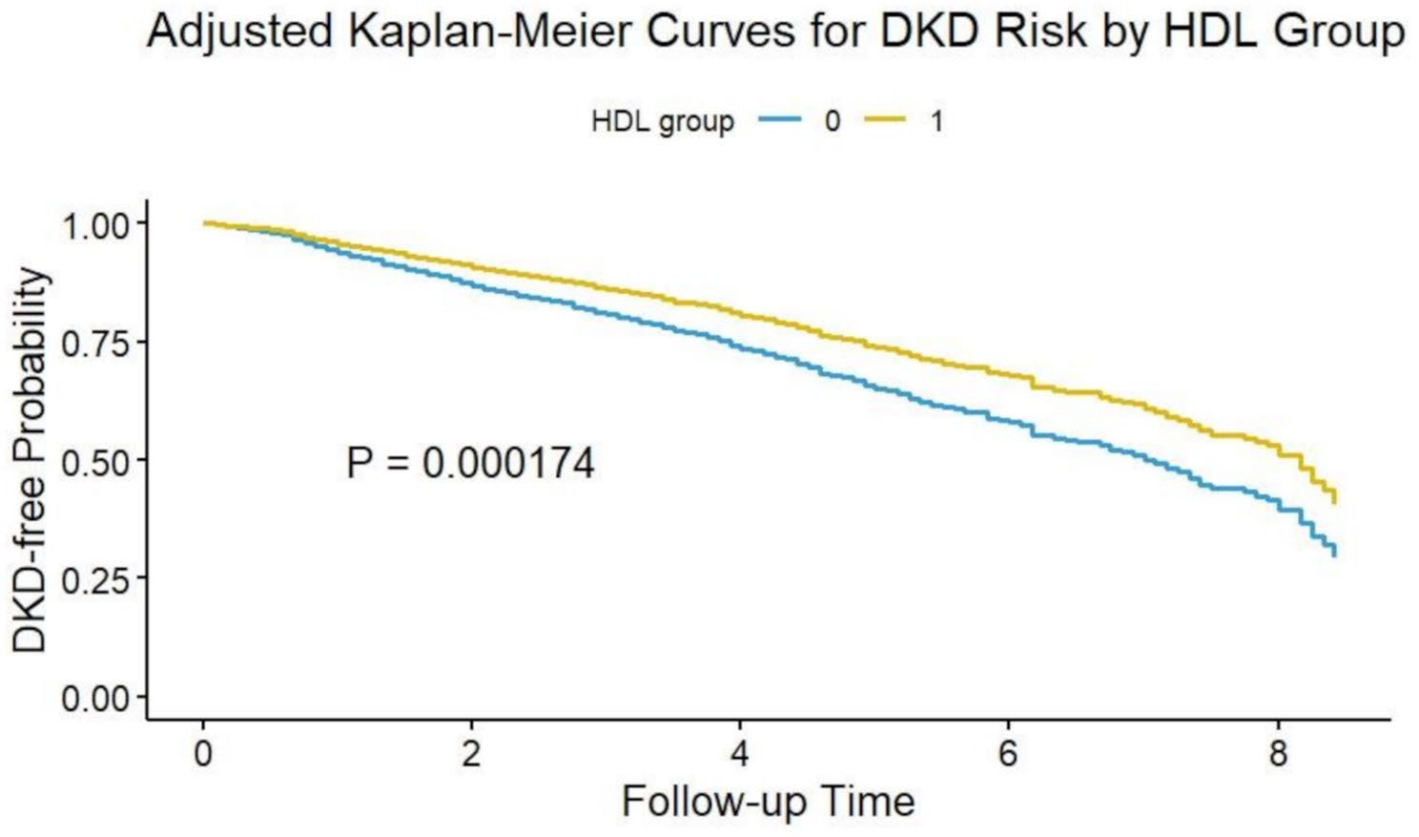
Adjusted Kaplan-Meier curves showed significantly reduced cumulative DKD incidence in the HDL-C ≥ 0.93 mmol/L group versus the lower HDL-C group in the overall cohort (*P* = 0.007). HDL group 0: subjects with HDL-C < 0.93 mmol/L; HDL group 1: subjects with HDL-C ≥ 0.93 mmol/L; DKD: diabetic kidney disease; HDL-C: high-density lipoprotein cholesterol

In the overall cohort, Kaplan-Meier curves (Fig. 5) showed that the cumulative incidence of DKD was significantly lower in the HDL-C ≥ 0.93 mmol/L group compared to the lower HDL-C group after adjusting for confounders (*P =* 0.007). In subgroups with hypertension, poor glycemic control, diabetes, BMI < 24 kg/m², and BMI > 28 kg/m², the cumulative DKD incidence was also significantly lower in those with HDL-C ≥ 0.93 mmol/L (*P <* 0.05). However, significant differences were detected in gender-based subgroups, with the HDL-C ≥ 0.93 mmol/L group having a significantly lower cumulative DKD incidence than the lower HDL-C group in males (Supplementary Figure S4A); however, no significant difference was found in females (Supplementary Figure S4B).

## Discussion

This large-scale longitudinal study provided novel evidence that the relationship between HDL-C and incident DKD risk was not uniform, exhibiting heterogeneity across clinically relevant subgroups. While HDL-C demonstrated an overall linear inverse association with DKD risk, this relationship manifested as distinct patterns of cumulative, threshold, and dual effects depending on sex, diabetes duration, and metabolic status. A cutoff of 0.93 mmol/L was identified as the optimal HDL-C threshold for DKD risk stratification, outperforming the conventional 1.0 mmol/L cutoff. Additionally, females derived continuous protection from increasing HDL-C levels without an apparent threshold dependency, whereas males exhibited threshold-dependent protection, suggesting sex-specific HDL functionality. The sex- and metabolic status-dependent effects of HDL-C could provide individualized targets for DKD prevention, potentially reconciling prior controversies in epidemiological studies. These findings challenge the ‘one-size-fits-all’ approach to HDL-C management for DKD prevention and underscore the necessity for subgroup-specific risk assessment strategies.

HDL-C not only reduces glomerular lipid deposition through reverse cholesterol transport but also inhibits renal injury through anti-inflammatory and antioxidant effects [23]– [24]. HDL-C can suppress the release of pro-inflammatory mediators like tumor necrosis factor-α and interleukin-6 while enhancing superoxide dismutase activity to alleviate oxidative stress, delaying glomerulosclerosis and tubulointerstitial fibrosis [23]. The protective effects of HDL-C were more pronounced in specific subgroups in this study, suggesting that its function may be modulated by the metabolic environment. In individuals with hypertension or obesity, HDL-C functionality may be impaired because of chronic inflammation or insulin resistance [25]. Nevertheless, the findings revealed significant threshold effects in these populations, implying that low HDL-C levels (< 0.93 mmol/L) could serve as an independent marker for DKD risk exacerbation under metabolic disturbances. HDL-C “quality” (such as particle size and apolipoprotein composition) may hold greater biological significance than absolute concentration [26], warranting future investigations incorporating functional assessments.

Compared with HbA1c, which has a well-established association with DKD risk, the absolute difference in HDL-C levels between DKD progressors and non-progressors was small (1.11 vs. 1.13 mmol/L). This apparent minor difference in the overall cohort might stem from the significant heterogeneity in the association patterns across the subgroups. Sex differences emerged as key determinants of HDL-C heterogeneity. In females, the continuous cumulative effect of HDL-C (HR = 0.41 per 1 mmol/L) was speculated to arise from the dual regulation of HDL. Estrogen has been reported to upregulate *APOA1* expression by activating the PPARα pathway in hepatocytes [27] and to inhibit NADPH oxidase activity, reducing oxidative damage to HDL caused by reactive oxygen species [28]. In contrast, a threshold effect (> 0.93 mmol/L, HR = 0.60) was observed in males, which might be due to their HDL being more susceptible to metabolic disturbances. A previous study indicated significant sex differences in HDL particle size, apolipoprotein composition, and functional activity [29]: female HDL is predominantly composed of large, apolipoprotein A1-enriched particles (HDL2b) that exhibit stronger antioxidant capacity and cholesterol efflux capacity, while males have a higher proportion of small, dense HDL3 particles; increased HDL3 levels may be associated with metabolic disturbances, and metabolic states exacerbate this heterogeneity. Obesity and insulin resistance induce HDL remodeling through oxidative stress, impairing anti-inflammatory functions by suppressing lecithin-cholesterol acyltransferase activity [25, 30]. In patients with diabetes, abnormalities in the HDL lipidome reduce reverse cholesterol transport efficiency [25]. These functional defects, independent of the HDL-C concentration, may cause males to compensate for insufficient functional HDL levels by maintaining a higher threshold.

The HDL-C threshold identified in this study (0.93 mmol/L) was lower than the 1.0 mmol/L recommended by traditional guidelines, but is explainable from pathophysiological and methodological perspectives. Mechanistically, low HDL-C levels (< 0.93 mmol/L) have been suggested to indicate severe lipid metabolism disorders, which act synergistically with insulin resistance and chronic inflammation to accelerate renal injury [31]. In contrast, hyperglycemia-induced glycation of apolipoprotein A1 inhibits its ability to activate lecithin-cholesterol acyltransferase, resulting in a 40% reduction in HDL cholesterol efflux efficiency in diabetic models [26]. Compared with individuals without diabetes, patients with diabetes are hypothesized to require higher HDL-C concentrations to compensate for functional impairments [32]. Because of this, a lower threshold may reflect these differences more sensitively. Methodologically, the threshold was determined using RCS and MSS and validated for robustness with bootstrap resampling. Although the 95% confidence interval was wide (0.60–2.20 mmol/L), the thresholds were densely distributed between 0.90 and 0.95 mmol/L and were close to the lower limit of the guidelines, indicating biological plausibility. Additionally, significantly fewer subgroups demonstrated protective effects when the traditional 1.0 mmol/L threshold was applied compared to the 0.93 mmol/L threshold, further supporting the necessity of adopting 0.93 mmol/L as an optimized target.

Subgroup analyses revealed a large effect of metabolic status on HDL-C levels. HDL-C levels showed completely divergent effects on the risk of DKD in populations with good versus poor glycemic control and were not significantly associated with DKD in the subgroup with well-controlled glycemia. This is potentially attributable to the preserved functional integrity of HDL under lower oxidative stress and inflammation levels. Improved glycemic control is often accompanied by the amelioration of other metabolic parameters, which might have synergistically reduced the risk of DKD, masking the independent effects of HDL-C. Although HDL-C concentrations remain normal or even elevated in patients with poor glycemic control, their function is severely impaired, and they fail to exert anti-inflammatory, antioxidant, or cholesterol efflux activities [26]. Under these conditions, the quantitative levels of HDL-C became a critical compensatory factor for qualitative deficiencies. Only by maintaining higher HDL-C concentrations could partial functional compensation occur through quantitative superiority, ultimately achieving statistical significance in DKD risk associations.

Duration-stratified analyses showed stronger threshold effects in patients with diabetes duration < 10 years (HR = 0.52 vs. ≥ 10 years: HR = 0.79), aligning with the “metabolic memory” phenomenon reported in the DCCT/EDIC study [33]. Early intensive glycemic control delays microvascular complications, whereas prolonged hyperglycemia may cause irreversible HDL functional damage [34]. When HDL-C fell below the threshold of 0.93 mmol/L, its function was insufficient to counteract renal injury from early metabolic disturbances (such as hyperglycemia or insulin resistance), manifesting as a pronounced threshold effect. However, in patients with long-duration diabetes with severely compromised HDL function, a single concentration threshold failed to reflect residual functionality, necessitating continuous accumulation of HDL-C levels to demonstrate renal protection.

In BMI-stratified analyses, patients with BMI > 28 kg/m² and < 24 kg/m² demonstrated significant protective effects against DKD, regardless of whether HDL-C was analyzed as a continuous or threshold variable, whereas the overweight group (BMI 24–28 kg/m²) showed no association. This may be related to the dynamic heterogeneity in metabolic status and HDL functional integrity. In the obese population, HDL function is severely impaired because of chronic inflammation and glycation modifications, necessitating a lower threshold to compensate for the reduced cholesterol efflux capacity [35, 36]. The continuous effect of HDL-C reflects a linear relationship between the concentration and the residual function. In non-obese individuals, stronger metabolic reserves (such as preserved insulin sensitivity) are associated with a higher proportion of HDL2b, enabling protection maintenance even at lower concentrations [37]. The overweight group was in a transitional stage between metabolic compensation and decompensation, where HDL particle heterogeneity (an increased proportion of small, dense subtypes) and confounding metabolic factors (such as a high TG/HDL-C ratio) diluted the functional protective effects, further weakening the statistical associations. These findings suggest that DKD risk assessment in overweight populations requires a comprehensive evaluation that incorporates lipid profiles and visceral fat distribution indicators.

### Study strengths and limitations

This study clarified HDL-C heterogeneity in DKD risk through methodological rigor and clinical stratification. The large longitudinal cohort of 3,040 patients provided robust statistical power to detect subgroup-specific associations. Additionally, comprehensive stratification by sex, diabetes duration, and metabolic status (hypertension, glycemic control, and obesity) revealed previously under-recognized heterogeneity in HDL-C effects, offering granular insights into populations benefiting from continuous protection (females) versus threshold-dependent risk reduction (males). These findings may advance epidemiological models by demonstrating that subgroup-specific associations reconcile the conflicting observations from previous studies. However, this study also had several limitations. The lack of HDL particle size measurements and functional indicators (such as cholesterol efflux capacity) prevented the determination of whether structural differences drove subgroup heterogeneity. Additionally, single-center recruitment and a relatively short median follow-up period (3.13 years) may have limited the generalizability of the findings to populations with different genetic/regional backgrounds or long-term outcomes. Despite the longitudinal design, dynamic adjustments were also technically challenging due to the retrospective design of the study; therefore, a time-dependent Cox regression analysis was not performed. This granularity deficit may have attenuated the temporal association between the changes in HDL-C levels and DKD progression. Additionally, residual confounding from unobserved factors (such as diet and exercise habits) could not be fully excluded despite rigorous adjustments. Lastly, this study did not perform a priori sample size calculations; instead, including all eligible patients who visited the hospital during the study period. Although this real-world approach enhances the generalizability of the findings, it may limit the statistical power to detect smaller effect sizes, particularly in subgroup analyses. In future studies, the HDL-C threshold (0.93 mmol/L), particularly its robust protective association in males and metabolically unstable subgroups, should be validated through multicenter studies. Additionally, mechanistic studies must integrate functional HDL metrics to determine whether this threshold represents a critical concentration of functional HDL particles capable of counteracting metabolic insults. Intervention trials should also target threshold-sensitive populations to test if elevating HDL-C > 0.93 mmol/L through lifestyle or pharmacotherapy replicates the risk reduction patterns observed in this cohort. These steps would address the limitations inherent to single-center retrospective designs and lay a methodological foundation for refining risk stratification in subgroups that exhibit threshold-dependent protection.

## Conclusions

This study revealed that the protective effects of HDL-C on DKD risk are not uniform, instead exhibiting sex- and metabolic context-dependent heterogeneity. Males and patients with diabetes duration < 10 years require maintaining HDL-C ≥ 0.93 mmol/L for threshold-dependent protection. Females benefit from cumulative HDL-C increases without threshold constraints, while metabolically unstable subgroups (hypertension, poor glycemic control, obesity) exhibit dual continuous and threshold effects. These findings may inform individualized risk stratification in specific populations, highlighting the need to consider subgroup characteristics rather than applying uniform HDL-C targets.

## Electronic supplementary material

Below is the link to the electronic supplementary material.

Supplementary Material 1

## Data Availability

The data underlying this article will be shared on reasonable request to the corresponding author.

## Abbreviations

ACEI: Angiotensin converting enzyme inhibitor
ARB: Angiotensin receptor blocker
ACR: Albumin-to-creatinine ratio; BMI: body mass index
DKD: Diabetes kidney disease; DBP: diastolic blood pressure
eGFR: Estimated glomerular filtration rate
GLP-1RA: Glucagon-like peptide-1 receptor agonists; HbA1c: glycated hemoglobin
HDL-C: High-density lipoprotein cholesterol; HR: Hazard ratios
LDL-C: Low-density lipoprotein cholesterol; MSS: maximum selected statistics
RCS: Restricted cubic spline; SBP: systolic blood pressure
SGLT2i: Sodium-glucose cotransporter-2 inhibitors; T2D: type 2 diabetes
