# Supplementary material for: Sex-specific and metabolic subgroup heterogeneity in high-density lipoprotein cholesterol associations with diabetic kidney disease risk: a retrospective cohort study
Source: Lipids Health Dis. 2025 Jun 7;24:205. doi: 10.1186/s12944-025-02632-4 (PMC12144804; doi:10.1186/s12944-025-02632-4)
Supplement: Supplementary file 1 — Supplementary Material 1 [file 12944_2025_2632_MOESM1_ESM.docx]

Figure S1. Determination of the optimal HDL-C threshold using the MSS method (A) and bootstrap validation (B) in the male subgroup. HDL-C: high-density lipoprotein cholesterol; MSS: maximum selected statistics.


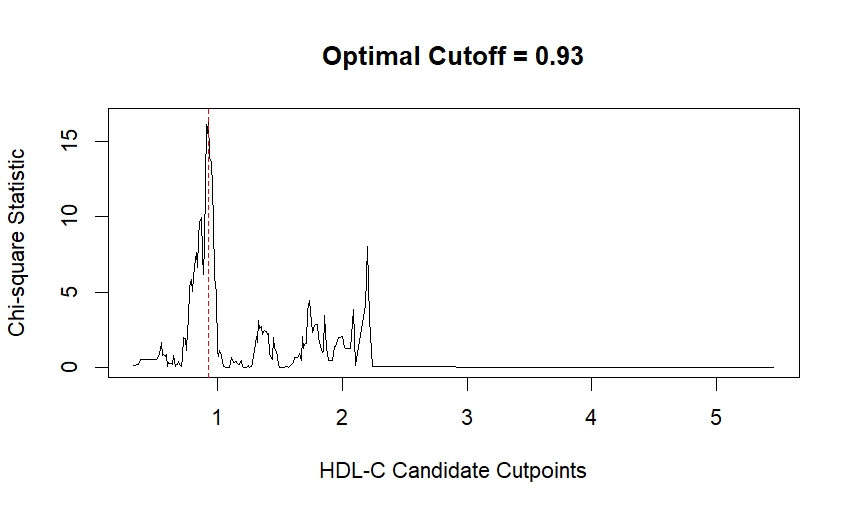

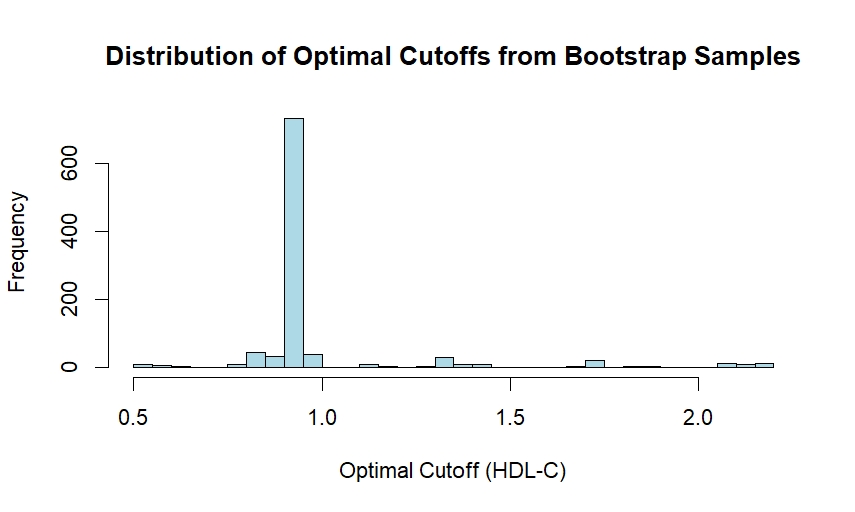


**A**

**B**


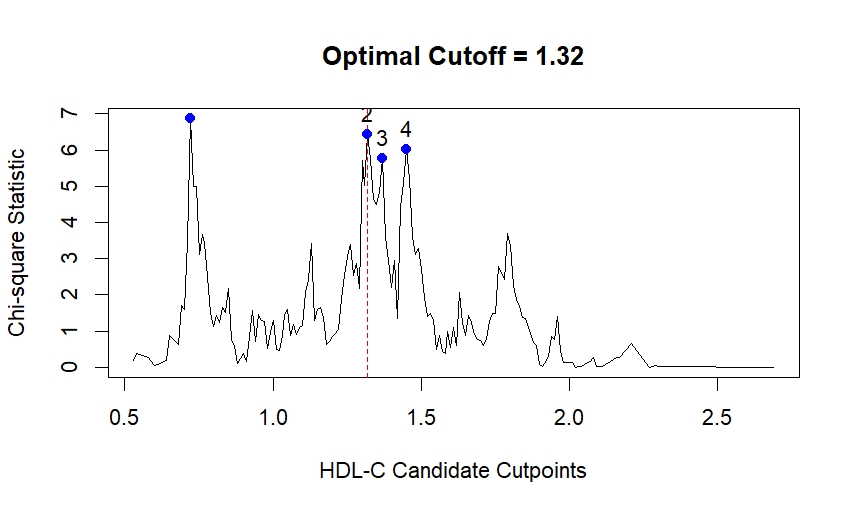

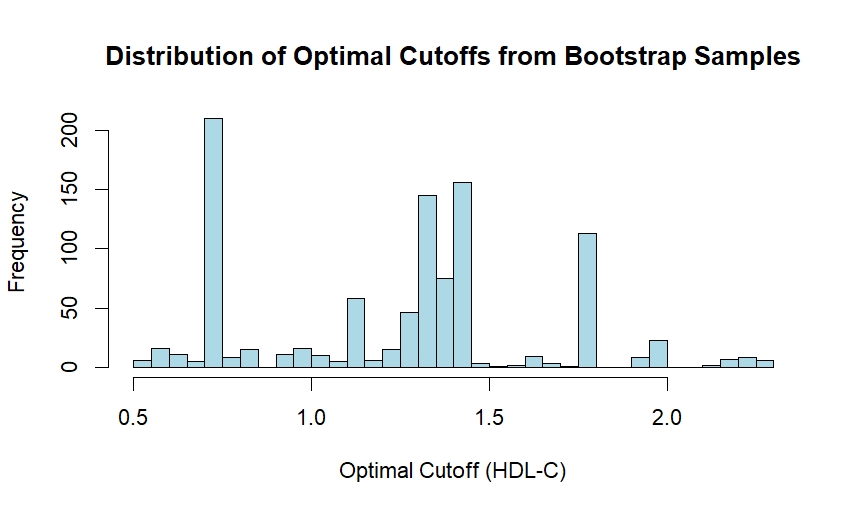


**A**

**B**

Figure S2. Determination of the optimal HDL-C threshold using the MSS method (A) and bootstrap validation (B) in the female subgroup. HDL-C: high-density lipoprotein cholesterol; MSS: maximum selected statistics.


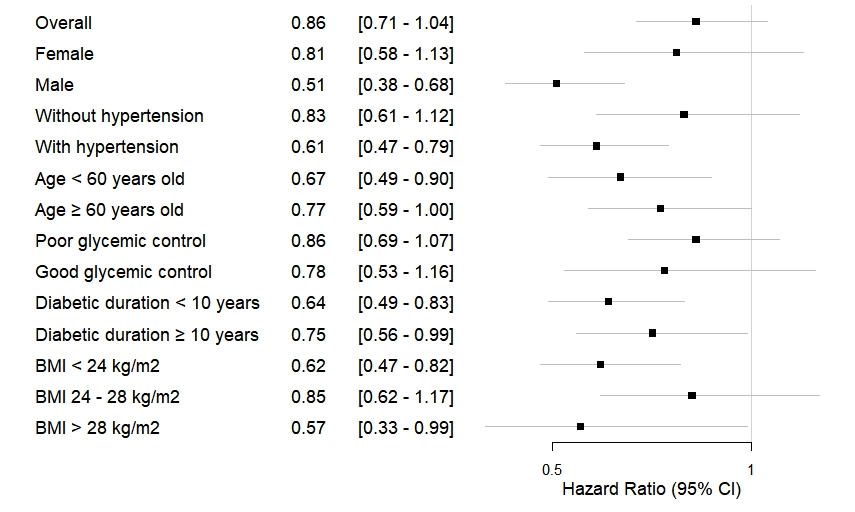
Figure S3. Sensitivity analysis with the traditional 1.0 mmol/L HDL-C cutoff confirmed subgroup heterogeneity in threshold effects and demonstrated superior validity of the 0.93 mmol/L threshold over the conventional cutoff for DKD risk stratification. DKD: diabetic kidney disease; HDL-C: high-density lipoprotein cholesterol; BMI: body mass index.

Figure S4. Gender-stratified Kaplan-Meier analysis demonstrated significantly lower cumulative DKD incidence in males with HDL-C ≥ 0.93 mmol/L compared to lower HDL-C groups (A), while no significant difference was observed in females (B). HDL group 0：subjects with HDL-C < 0.93 mmol/L; HDL group 1：subjects with HDL-C ≥ 0.93 mmol/L; DKD: diabetic kidney disease; HDL-C: high-density lipoprotein cholesterol.


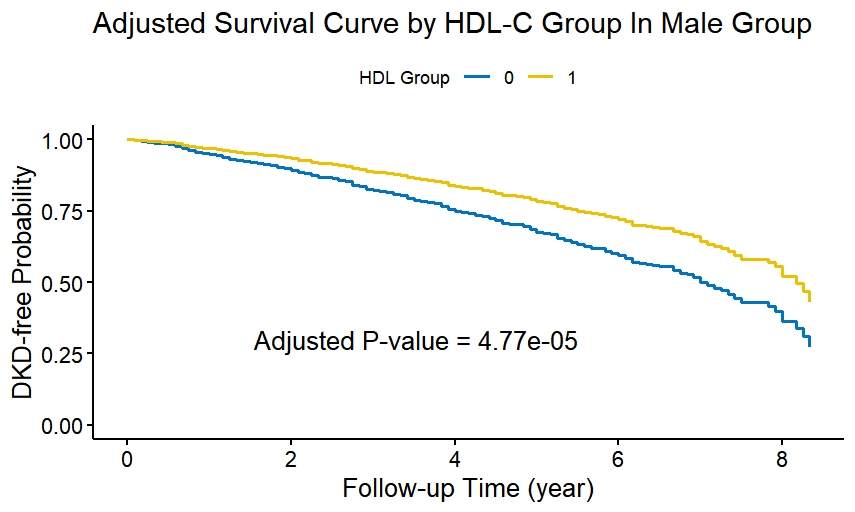

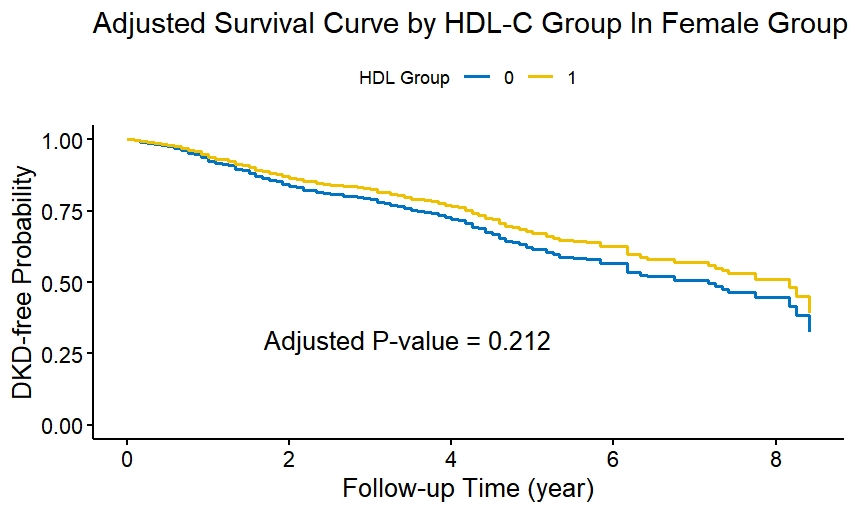


**B**

**A**
